# Clinical outcomes of one-hole split endoscope versus unilateral biportal endoscopy for lumbar degenerative diseases

**DOI:** 10.3389/fsurg.2026.1915621

**Published:** 2026-10-19

**Authors:** Cheng Li, Yifei Huang, Yuanyuan Dou, Muhaimaiti Abudurezhake, Lin Li, Qiucheng Chen, Kun Wang, Hailong Wang, Wu Xiaoye, Zhanjun Ma

**Affiliations:** 1 XinJiang Medical University, Urumqi, China; 2 Xinjiang Medical University Affiliated Fourth Hospital, Urumqi, China

**Keywords:** clinical outcomes, lumbar degenerative diseases, minimally invasive spine surgery, one-hole split endoscope, oswestry disability index, perioperative outcomes, unilateral biportal endoscopy, visual analog scale

## Abstract

**Objective:**

This study aims to compare the clinical efficacy and safety of the novel One-hole Split Endoscope (OSE) technique with the established Unilateral Biportal Endoscopy (UBE) technique for the treatment of lumbar degenerative diseases (LDD).

**Methods:**

A retrospective cohort study was conducted from January 2021 to December 2023, involving 116 patients with LDD. Patients were allocated to either the OSE group (*n* = 60) or the UBE group (*n* = 56). Perioperative outcomes (operative time, blood loss, hospital stay, incision length), clinical scores (VAS for back/leg pain, ODI), and complication rates were systematically compared between the two groups.

**Results:**

Both groups demonstrated significant clinical improvement postoperatively. The OSE procedure required a longer operative time (135.8 ± 12.6 min vs. 102.4 ± 10.9 min, *P* < 0.001). However, the OSE group demonstrated advantages in several key perioperative metrics, including a significantly shorter hospital stay (3.9 ± 0.8 days vs. 5.4 ± 1.1 days, *P* < 0.001) and a smaller incision length (1.5 ± 0.2 cm vs. 2.7 ± 0.3 cm, *P* < 0.001). Intraoperative blood loss was minimal and comparable between the two groups (*P* > 0.05). The OSE group also showed earlier and superior functional recovery, evidenced by significantly lower ODI scores at 1-week, 1-month, and final follow-up (*P* < 0.05), whereas no significant differences were found in postoperative pain VAS scores or in the overall complication rate (*P* = 0.127).

**Conclusion:**

Both OSE and UBE techniques are effective and safe for treating LDD. Despite a longer operative time, the OSE technique offers distinct advantages in minimizing surgical trauma, promoting faster recovery, and achieving better early functional outcomes. OSE represents a valuable and innovative minimally invasive option in the spine surgeon's arsenal.

## Introduction

Lumbar degenerative disease (LDD) is a common cause of low back and leg pain in middle-aged and elderly populations, characterized by progressive degeneration involving multiple structures such as intervertebral discs, facet joints, and ligaments. With the accelerating global aging trend and changes in modern lifestyle, the prevalence of LDD continues to rise. This condition not only causes pain and functional impairment but also leads to substantial healthcare expenditures and loss of social productivity, making it a significant public health concern (1, 2). The natural course of LDD typically progresses through three stages—dysfunctional stage, unstable stage, and stabilization stage—reflecting a complex process of gradual biomechanical imbalance and structural remodeling in the lumbar spine (3).

In terms of treatment, conservative management is suitable for patients with early or mild symptoms. However, surgical intervention becomes necessary for those who do not respond to conservative treatment or present with progressive neurological deficits (4). In recent years, spinal surgery has evolved toward minimally invasive and precision techniques. Since the 1990s, percutaneous spinal endoscopy has advanced rapidly. The YESS technique introduced by Yeung et al. and the TESSYS technique popularized by Hoogland et al. significantly improved outcomes in the treatment of lumbar disc herniation via the transforaminal approach (5). Nevertheless, conventional microscope-assisted surgery carries risks such as high rates of cerebrospinal fluid leakage and difficulty in controlling intraoperative bleeding (4), while early percutaneous transforaminal endoscopy faced limitations due to its coaxial operating channel and restricted instrument mobility, posing challenges in managing complex spinal stenosis and bony pathologies (6).

To overcome these limitations, Unilateral Biportal Endoscopy (UBE) emerged accordingly. This technique establishes independent viewing and working channels combined with continuous saline irrigation, maintaining minimally invasive advantages while significantly improving visual clarity and operational flexibility. It is particularly suitable for long-segment decompression and highly migrated disc lesions (7). Multiple clinical studies have demonstrated that UBE excels in controlling intraoperative bleeding, preserving paraspinal muscles, and promoting postoperative functional recovery (8). In recent years, Professor Zhu Tengyue proposed the One-hole Split Endoscope (OSE) technique, further enriching the spectrum of minimally invasive spinal endoscopy. OSE employs a single incision with separated viewing and working channels, combining the minimal tissue trauma of coaxial systems with the maneuverability of biportal techniques, showing unique advantages in addressing pathologies within the intervertebral space and facet joint regions (9, 10).

Although preliminary studies suggest favorable early outcomes with OSE, its clinical application remains relatively recent both domestically and internationally, with most published reports involving small sample sizes. There is a lack of systematic comparative studies against the established UBE technique. Therefore, this study aims to conduct a retrospective cohort study comparing OSE and UBE in the treatment of LDD, focusing on perioperative indicators, pain and functional improvement, and complication rates. The goal is to clarify the efficacy and safety of OSE, provide evidence-based support for its rational clinical application and promotion, and further advance individualized and precise minimally invasive treatment for lumbar degenerative diseases.

## Materials and methods

### General information

This retrospective cohort study was conducted at the Xinjiang Medical University Affiliated fourth Hospital from January 2021 to December 2023.

#### Inclusion criteria

1. Presence of low back pain with radiating lower limb pain or numbness;
2. Corresponding symptomatic evidence on MRI and computed tomography (CT);
3. Failure of or worsening symptoms after 12 weeks of structured conservative treatment (including regular traction, massage, physical therapy, and traditional Chinese medicine), significantly affecting work and daily life;
4. No previous history of lumbar spine surgery.

#### Exclusion criteria

1. Lumbar spondylolisthesis greater than Meyerding grade I or lumbar instability;
2. History of spinal scoliosis, spinal infection, spinal tumor, spinal tuberculosis, or hip/knee osteoarthritis;
3. Combined severe spinal deformity or diseases that may cause muscle injury (e.g., myositis, trauma, etc.);
4. Severe cardiovascular or cerebrovascular diseases, coagulation disorders, or other surgical contraindications (including severe liver/kidney disease, pulmonary disease, mental disorders, etc.).

A total of 116 patients were enrolled and divided into two groups according to the surgical approach: the OSE group (*n* = 60) and the UBE group (*n* = 56). The cohort consisted of 64 cases of lumbar disc herniation and 52 cases of lumbar spinal stenosis. Ethical approval for this retrospective study was obtained from the Institutional Review Board, which waived the requirement for written informed consent due to the nature of the study.

In this non-randomized study, the choice of surgical technique was based on a comprehensive evaluation of the patient's pathological anatomy and the surgeon's assessment. OSE was generally preferred for patients with single-level disc herniation or unilateral lateral recess stenosis, while UBE was more frequently selected for cases with central canal stenosis or bilateral decompression needs. All surgical procedures were performed by a single senior surgeon who had extensive experience in minimally invasive spine surgery (over 500 cases of UBE) to minimize the confounding effects of the initial learning curve.

### Surgical procedure

UBE group: The patient was placed in a prone position under general anesthesia with endotracheal intubation. The target level was confirmed via preoperative imaging localization, and two incision lines, each measuring 1.0–1.5 cm, were marked on the body surface corresponding to the upper and lower aspects of the relevant intervertebral space. The right incision (1.5 cm) served as the main working portal, while the left incision (1.0 cm) functioned as the viewing portal. Following routine disinfection and draping, the skin and subcutaneous tissues were incised layer by layer. Bilateral working paths were established using sequential dilators. The arthroscopy system was introduced through the viewing portal, and radiofrequency ablation instruments were placed via the working portal. Soft tissues at the lamina-spinous process junction were first cleared. A drill was used to trim the superior edge of the lower lamina, the inferior edge of the upper lamina, and the medial one-third of the facet joint bone. The ligamentum flavum was then sharply dissected and resected. The decompression area was extended laterally along the course of the nerve root to fully expose the dural sac and nerve root. In cases with concurrent disc herniation, nucleotomy was performed simultaneously. Subsequently, the contralateral structures were addressed: the base of the spinous process was drilled away, and the contralateral ligamentum flavum along with the medial edge of the superior articular process were removed to achieve bilateral nerve root canal expansion. After confirming under endoscopy that the dural sac was free from compression and the nerve roots were relaxed, hemostasis was achieved, the surgical field was irrigated with saline, a drainage tube was placed, and the wound was closed in layers.

OSE group: Under general anesthesia with orotracheal intubation, the patient was positioned prone on a radiolucent table with appropriate hip and knee flexion. After fluoroscopically identifying and marking the left L4/5 interlaminar window, a 15 mm incision was made following sterile preparation. Sequential dilators were introduced toward the L4 lamina-spinous process junction under fluoroscopic guidance. With the endoscope positioned and continuous saline irrigation maintained, soft tissue was cleared using pituitary forceps and radiofrequency ablation to expose the ligamentum flavum. Partial laminectomy of the left L4 and L5 laminae was performed, followed by careful resection of the ligamentum flavum to visualize the dura and L5 nerve root. The herniated disc fragment compressing the nerve root was identified and removed with pituitary rongeurs. After confirming complete decompression and achieving hemostasis, a gelatin sponge was placed over the dura, and the wound was closed in layers.

All procedures were performed via the left-sided approach unless contraindicated by the pathology.

### Postoperative treatment

Postoperative care included 24–48 h of prophylactic antibiotics, neuroprotective/analgesic medications, and early ambulation protocols. Patients received structured education on spinal biomechanics and ERAS-compliant pain control. Drains were removed if output was <50 mL/24 h, with a soft lumbar brace worn for 3 months. Activity restrictions prohibited heavy lifting or intense lumbar movements for 6 months.

### Observational indicators

Data were collected through the hospital's electronic medical record system. The following parameters were documented:

1. Demographic data: age, gender, body mass index (BMI), surgical level, underlying chronic conditions (including hypertension, diabetes, coronary heart disease, endocrine disorders, metabolic diseases, and arthritis), smoking history, and alcohol consumption.
2. Surgical parameters: intraoperative blood loss (estimated by the volume of blood in the suction container minus the amount of irrigation fluid used, combined with the weight gain of surgical gauzes, where 1 g ≈ 1 mL), operative time (from skin incision to skin closure), postoperative hospital stay (days from surgery to discharge), hospitalization costs (total inpatient expenses), and incision length (measured using a sterile ruler at the time of skin closure).
3. Clinical outcomes: visual analogue Scale (VAS) scores for low back and leg pain, and Oswestry Disability Index (ODI) scores, assessed preoperatively and at 1 week, 1 month, 6 months, and at the final follow-up (average 14 months).
4. Complications: all intraoperative and postoperative complications were recorded.

### Statistical analysis

All data were analyzed with SPSS 26.0 (IBM Corp). Continuous variables were examined for normality using the Kolmogorov–Smirnov test. For comparing these variables between groups, the independent samples t-test was applied to normally distributed data, and the Mann–Whitney *U* test was used for non-normally distributed data. For longitudinal data (VAS and ODI scores) measured at multiple time points (preoperative, 1 week, 1 month, and final follow-up), we used a linear mixed-effects model with an unstructured covariance matrix. The model included fixed effects for time, group, and the time-by-group interaction, with subjects treated as random effects. When a significant group-by-time interaction was detected, *post-hoc* pairwise comparisons were performed using the Bonferroni correction. Independent t-tests were used for cross-sectional comparisons at individual time points, as presented in Table 3. To adjust for potential confounding variables in this non-randomized study, a multivariate linear regression analysis was performed for the primary outcome measures (ODI score at final follow-up and hospital stay). Variables with a *P* value < 0.10 in univariate analysis or those considered clinically relevant (age, sex, BMI, diagnosis type, and surgical segment) were entered into the multivariate model. Results are presented as adjusted *β* coefficients with 95% confidence intervals. Associations involving categorical variables were evaluated with the chi-square test or Fisher's exact test, as appropriate. A two-tailed *P* < 0.05 was set as the threshold for statistical significance.

### Sample size and power analysis

A *post-hoc* power analysis was performed using G*Power 3.1. Based on the observed difference in ODI scores at final follow-up (mean difference = 1.9, pooled SD = 3.05) and a sample size of 116 (60 in OSE group, 56 in UBE group), with *α* = 0.05 (two-tailed), the achieved power for detecting this difference was 0.82, exceeding the conventional threshold of 0.80. For the primary perioperative outcomes (hospital stay and incision length), the achieved power exceeded 0.99. This indicates that the sample size was adequate to detect clinically meaningful differences in the main outcome measures.

## Results

### The comparison of general data

This study retrospectively enrolled 116 patients diagnosed with lumbar degenerative disease, who were assigned to two surgical groups according to the technique employed: the OSE group (*n* = 60) and the UBE group (*n* = 56). All participants completed the study protocol, and no dropouts were recorded (Figure 1). Baseline demographic and clinical characteristics were comparable between the two groups, with no statistically significant differences observed (all *P* > 0.05), as detailed in Table 1.

**Figure 1:**
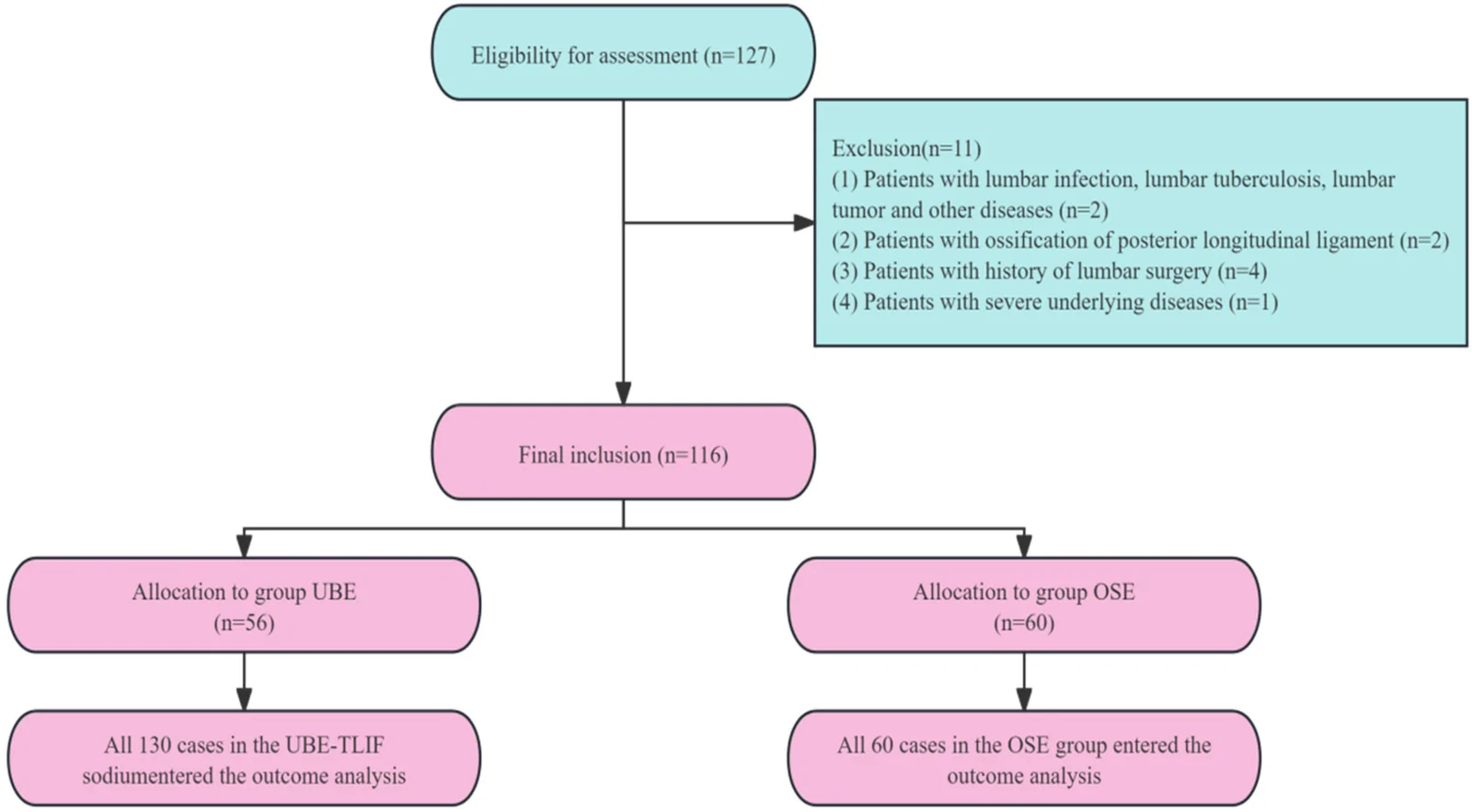
Flow chart of test grouping.

**Table 1:** Comparison of preoperative baseline data between the Two groups.

Index	OSE (*n* = 60)	UBE (*n* = 56)	*χ²/t*	*P*
Age (years, x¯ ± s)	58.7 ± 8.9	57.3 ± 9.6	0.806	0.422
Gender (*n*, Male/Female)	26/34	29/27	0.873	0.350
BMI (kg/m², x¯ ± s)	25.3 ± 3.2	25.8 ± 3.7	−0.773	0.441
Surgical Segment, *n* (%)			1.325	0.516
L3/4	4 (6.7%)	2 (3.6%)		
L4/5	48 (80.0%)	46 (82.1%)		
L5/S1	8 (13.3%)	8 (14.3%)		
Comorbidities, *n* (%)				
Hypertension	18 (30.0%)	16 (28.6%)	0.031	0.860
Diabetes	12 (20.0%)	15 (26.8%)	0.801	0.371
Coronary Heart Disease	7 (11.7%)	5 (8.9%)	0.248	0.619
Diagnosis, *n* (%)			0.162	0.922
Lumbar Disc Herniation	34 (60.0%)	30 (55.4%)		
Lumbar Spinal Stenosis	26 (40.0%)	26 (44.6%)		
Follow-up (months, x¯ ± s)	14.2 ± 2.1	13.8 ± 2.4	0.957	0.341

Data are presented as mean ± standard deviation or number (percentage). BMI: body mass index. Continuous variables were analyzed using independent samples t-test; categorical variables were analyzed using chi-square test.

### Comparison of surgical related indicators

All patients successfully underwent their assigned procedures. Comparative analysis revealed that the OSE group required a significantly longer operative time compared to the UBE group (135.8 ± 12.6 min vs. 102.4 ± 10.9 min, *P* < 0.001). However, the OSE group was associated with a significantly shorter postoperative hospital stay (3.9 ± 0.8 days vs. 5.4 ± 1.1 days, *P* < 0.001) and a smaller incision length (1.5 ± 0.2 cm vs. 2.7 ± 0.3 cm, *P* < 0.001). Notably, both groups exhibited minimal and comparable intraoperative blood loss (22.5 ± 5.3 mL vs. 20.8 ± 4.9 mL, *P* = 0.073) and hospitalization costs (32,485 ± 4,210 CNY vs. 34,180 ± 5,360 CNY, *P* = 0.068), with no statistically significant differences between them. These detailed results are presented in Table 2.

**Table 2:** Comparison of perioperative outcomes between the Two groups.

Index	OSE (*n* = 60)	UBE (*n* = 56)	*t*	*P*
Operative Time (min)	135.8 ± 12.6	102.4 ± 10.9	15.384	<0.001
Blood Loss (mL)	22.5 ± 5.3	20.8 ± 4.9	1.812	0.073
Hospital Stay (days)	3.9 ± 0.8	5.4 ± 1.1	−8.273	<0.001
Incision Length (cm)	1.5 ± 0.2	2.7 ± 0.3	−24.583	<0.001
Hospitalization Costs (CNY, *x¯* *±* *s*)	32,485 ± 4,210	34,180 ± 5,360	−1.843	0.068

Data are presented as mean ± standard deviation. All comparisons were performed using independent samples t-test. Blood loss was estimated using the combined method of suction container volume (minus irrigation fluid) and gauze weight change (1 g ≈ 1 mL). Incision length was measured with a sterile ruler at skin closure.

### Comparison of VAS and ODI before and after surgery

No statistically significant differences were observed in the preoperative VAS scores for back pain and leg pain, nor in the ODI scores, between the two groups (*P* > 0.05). Postoperatively, both groups showed significant clinical improvement at all follow-up time points compared to their preoperative baselines. While the intergroup comparison of VAS scores at various postoperative intervals showed no significant differences, the OSE group exhibited significantly lower ODI scores at the 1-week, 1-month, and final follow-up assessments compared to the UBE group (*P* < 0.05). Comprehensive data are summarized in Table 3 and Figure 2.

**Table 3:** Comparison of VAS and ODI scores before and after surgery (x¯ ± s).

Index	Follow-up	OSE (*n* = 60)	UBE (*n* = 56)	*t*	*P*
VAS (Back)	Pre-op	7.25 ± 1.32	7.18 ± 1.41	0.273	0.785
	1 Week	3.62 ± 0.95^a^	3.88 ± 1.05^a^	−1.384	0.169
	1 Month	2.25 ± 0.76^b^	2.48 ± 0.85^b^	−1.489	0.139
	6 Months	1.68 ± 0.72^c^	1.92 ± 0.78^c^	−1.697	0.092
	Final follow-up	1.32 ± 0.68^d^	1.55 ± 0.74^d^	−1.714	0.089
VAS (Leg)	Pre-op	6.75 ± 1.48	6.82 ± 1.53	−0.245	0.807
	1 Week	2.98 ± 1.15^a^	3.25 ± 1.28^a^	−1.169	0.245
	1 Month	1.75 ± 0.82^b^	1.98 ± 0.91^b^	−1.384	0.169
	6 Months	1.12 ± 0.58^c^	1.38 ± 0.64^c^	−1.814	0.072
	Final follow-up	0.95 ± 0.65^d^	1.18 ± 0.72^d^	−1.778	0.078
ODI (%)	Pre-op	65.8 ± 8.7	67.2 ± 9.1	−0.824	0.412
	1 Week	36.4 ± 6.8^a^	39.2 ± 7.1^a^	−2.104	0.038
	1 Month	20.8 ± 5.2^b^	23.1 ± 5.6^b^	−2.218	0.029
	6 Months	14.5 ± 3.8^c^	17.2 ± 4.1^c^	−3.612	<0.001
	Final follow-up	11.2 ± 2.9^d^	13.1 ± 3.2^d^	−3.273	0.001

Data are presented as mean ± standard deviation. The values in the table represent the results of cross-sectional comparisons between the two groups at each individual time point, analyzed using independent samples t-tests.

a *P* < 0.05 vs. Pre-op.

b *P* < 0.05 vs. 1 Week.

c *P* < 0.05 vs. 1 Month.

d *P* < 0.05 vs. 6 Months.

To evaluate the overall longitudinal trends, a linear mixed-effects model was additionally applied with Bonferroni correction for multiple comparisons. The analysis revealed a significant main effect of time for all scores (*P* < 0.001), confirming the overall efficacy of both techniques. A significant group-by-time interaction was found for ODI scores (*F* = 3.92, *P* = 0.012), indicating that the OSE group experienced faster functional recovery over time compared with the UBE group. No significant group-by-time interaction was observed for VAS back pain (*F* = 1.23, *P* = 0.301) or VAS leg pain (*F* = 1.56, *P* = 0.201). The main effect of group was not significant for any outcome measure (*P* > 0.05 for all), suggesting that the overall magnitude of improvement was comparable between groups, but the trajectory of recovery differed for ODI.

**Figure 2:**
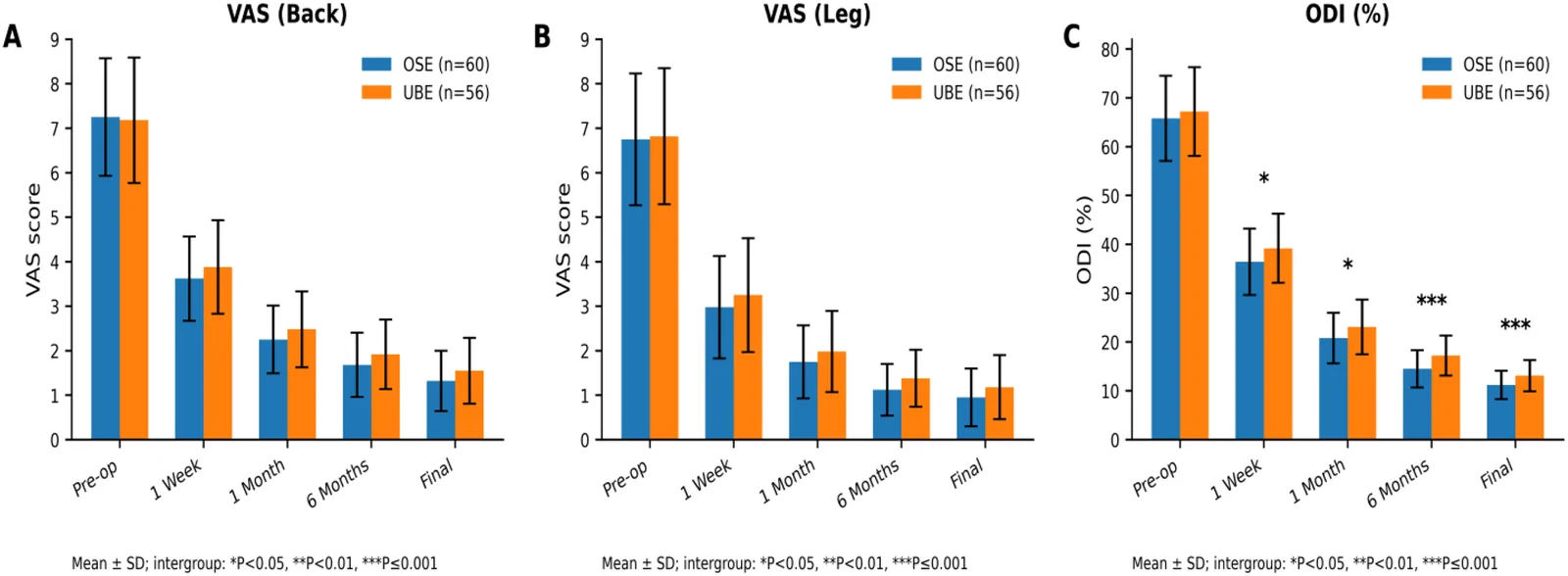
Longitudinal trends of VAS and ODI scores in the OSE and UBE groups. **(A)** VAS for back pain; **(B)** VAS for leg pain; **(C)** ODI scores. Data are presented as mean ± standard deviation. OSE, One-hole Split Endoscope; UBE, Unilateral Biportal Endoscopy; VAS, Visual Analog Scale; ODI, Oswestry Disability Index. *P < 0.05 for within-group comparisons vs. preoperative values at all follow-up time points. The OSE group showed significantly lower ODI scores at 1 week, 1 month, 6 months, and final follow-up compared with the UBE group (*P* < 0.05).

To address the potential heterogeneity of the included pathologies, we performed an exploratory descriptive analysis stratified by diagnosis. In both the lumbar disc herniation and lumbar spinal stenosis subgroups, the OSE technique consistently demonstrated trends similar to the main analysis: a longer operative time but a shorter hospital stay and lower ODI scores at the final follow-up compared to UBE. However, due to the limited sample size within each subgroup after stratification, formal statistical testing for interaction was not performed, and these descriptive findings should be interpreted with caution.

### Multivariate analysis of potential confounding factors

To further validate the independent effect of the surgical technique on clinical outcomes, multivariate linear regression was performed. After adjusting for age, sex, BMI, diagnosis type, and surgical segment, the OSE technique remained independently associated with a significantly shorter hospital stay and lower ODI scores at the final follow-up. Detailed results are presented in Table 4. These findings confirm that the observed advantages of OSE are not attributable to baseline imbalances between the two groups.

**Table 4:** Multivariate linear regression analysis for primary outcomes.

Outcome Variable	Predictor (OSE vs. UBE)	Adjusted *β*	95% CI	*P*
Hospital Stay (days)	OSE	−1.35	−1.72 to −0.98	<0.001
ODI at Final Follow-up (%)	OSE	−1.89	−2.97 to −0.81	0.001

Adjusted for age, sex, BMI, diagnosis type (LDH vs. LSS), and surgical segment. β, regression coefficient; CI, confidence interval; ODI, Oswestry Disability Index; OSE, One-hole Split Endoscope; UBE, Unilateral Biportal Endoscopy; LDH, lumbar disc herniation; LSS, lumbar spinal stenosis.

### Comparison of complications

Analysis of postoperative complications showed that the overall complication rate was 8.3% (5/60) in the OSE group and 17.9% (10/56) in the UBE group. The spectrum of complications included dural tear, postoperative radiculitis, epidural hematoma, and superficial infection. No severe complications such as neurological or vascular injuries were recorded in either cohort. Statistical analysis determined that there were no significant differences in the incidence rates of individual complications or in the overall complication rate between the two groups (*P* > 0.05). A complete breakdown is provided in Table 5.

**Table 5:** Comparison of complication rates between the Two groups [n (%)].

Complication	OSE (*n* = 60)	UBE (*n* = 56)	*χ²*	*P*
Dural Tear	1 (1.7%)	2 (3.6%)	0.404	0.525
Postoperative Radiculitis	2 (3.3%)	4 (7.1%)	0.905	0.341
Epidural Hematoma	1 (1.7%)	2 (3.6%)	0.404	0.525
Superficial Infection	1 (1.7%)	2 (3.6%)	0.404	0.525
Overall Rate	5 (8.3%)	10 (17.9%)	2.333	0.127

Data are presented as number (percentage). All comparisons were performed using chi-square test or Fisher's exact test as appropriate.

### Typical cases

Figures 3A–F represent the OSE group. The patient is a 52-year-old female with a 3-year history of low back pain, accompanied by bilateral lower limb numbness and radiating pain for 6 months, and lumbar spinal stenosis at L4-L5. Figure 3A: Preoperative anteroposterior x-ray; Figure 3B: Preoperative lateral x-ray; Figure 3C: Preoperative MRI; Figure 3D: postoperative orthosis x-ray; Figure 3E: Postoperative lateral x-ray; Figure 3F: Postoperative MRI. Figure 3G: Intraoperative endoscopic view showing the decompressed dural sac and exiting nerve root after removal of the ligamentum flavum.

**Figure 3:**
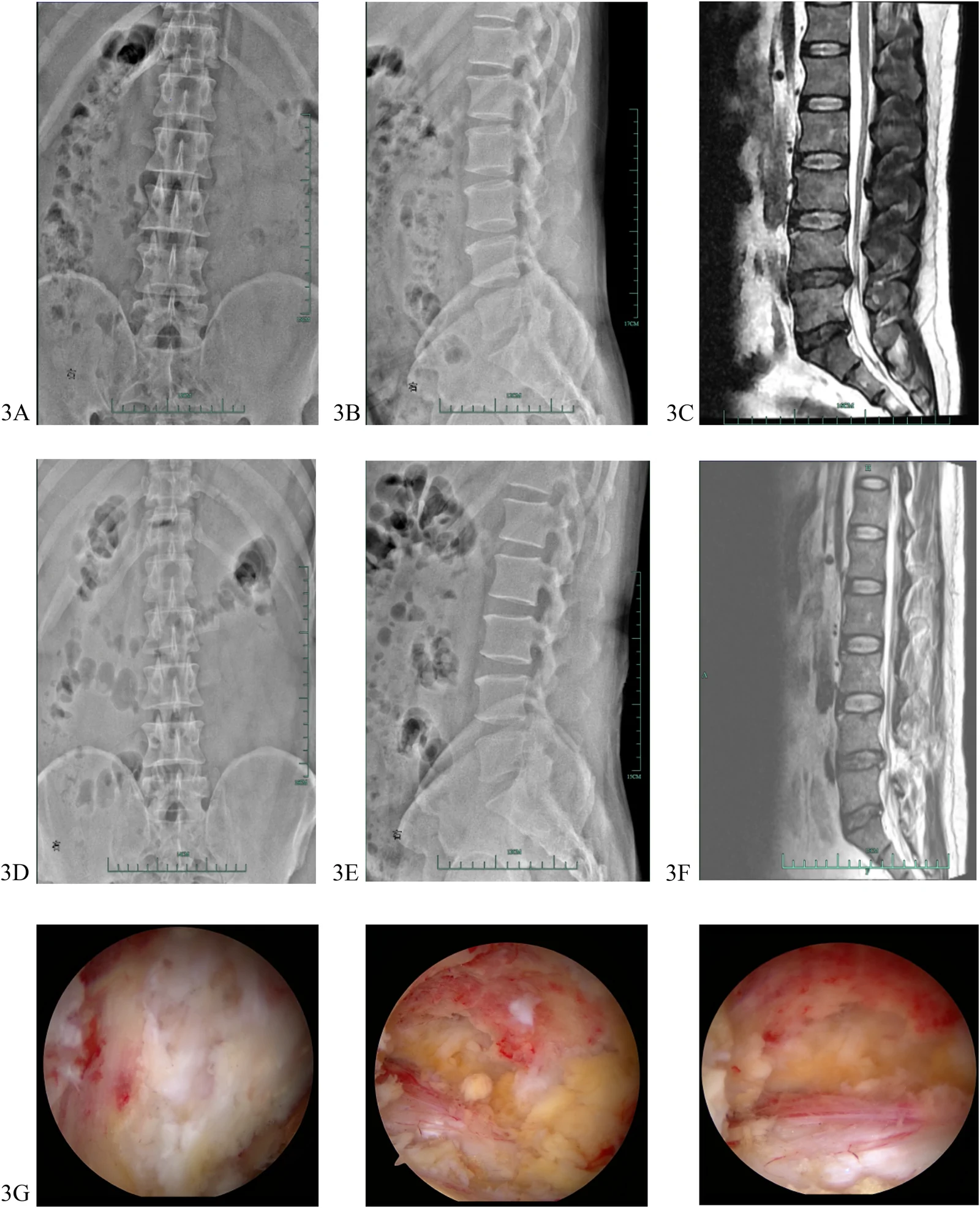
**(A)** Preoperative anteroposterior x-ray; **(B)** Preoperative lateral x-ray; **(C)** Preoperative MRI; **(D)** postoperative orthosis x-ray; **(E)** Postoperative lateral x-ray; **(F)** Postoperative MRI. **(G)** Intraoperative endoscopic view showing the decompressed dural sac and exiting nerve root after removal of the ligamentum /avum.

Figures 4A–F represent the UBE group. The patient is a 36-year-old female with progressive low back pain and intermittent claudication for 2 years, which significantly worsened over the past 3 months, and lumbar disc herniation at L4-L5.

**Figure 4:**
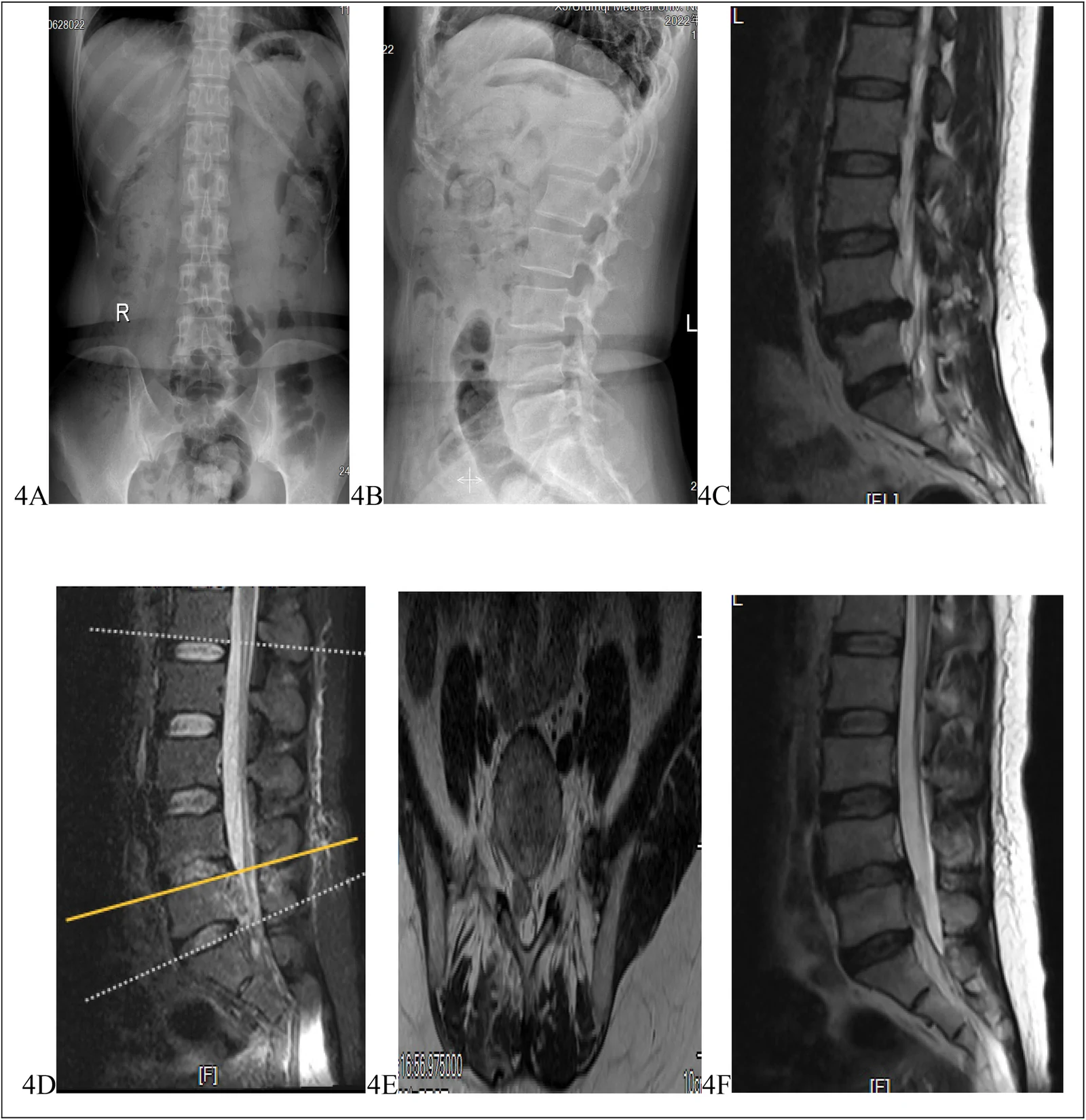
**(A)** Preoperative anteroposterior x-ray; **(B)** Preoperative lateral x-ray; **(C)** Preoperative MRI; **(D,E)** Postoperative CT scans; **(F)** Postoperative MRI.

Figure 4A: Preoperative anteroposterior x-ray; Figure 4B: Preoperative lateral x-ray; Figure 4C: Preoperative MRI; Figures 4D,E: Postoperative CT scans; Figure 4F: Postoperative MRI.

## Discussion

Minimally invasive surgical treatment for LDD has seen significant advances in recent years, with UBE established as a mature and reliable option (11). The OSE technique, as an emerging technology, aims to integrate the viewing and working channels through a single incision, seeking to further reduce surgical trauma while preserving the flexibility of UBE (12). This retrospective cohort study systematically compared the efficacy and safety of OSE vs. UBE for LDD, finding that while both techniques effectively alleviated symptoms, OSE demonstrated unique advantages in promoting faster recovery and superior early functional outcomes.

A key finding of this study reveals distinct perioperative profiles for the two techniques. The most notable difference was the significantly longer operative time required for the OSE group compared to the UBE group. This finding aligns with some initial reports (13–15), and we attribute it to the following reasons: as a novel technique, OSE is likely associated with a learning curve that has not yet been fully overcome. Although all OSE procedures in this study were performed by a single senior surgeon highly experienced in UBE, the inherent differences in instrument coordination within a single working channel still present a technical challenge, contributing to the prolonged operative time. Future studies should investigate how this operative time decreases as surgeons progress through their learning curves for OSE (16). However, it is crucial to emphasize that the longer operative time did not translate into greater surgical trauma or risk. On the contrary, the OSE group excelled in key metrics reflecting minimal invasiveness. First, intraoperative blood loss was minimal and comparable between the two groups (approximately 20 mL), with no statistical difference. This confirms that the OSE technique is equally effective as UBE in terms of continuous irrigation and precise radiofrequency haemostasis (17). More importantly, the OSE group benefited from a significantly shorter postoperative hospital stay and a markedly smaller incision length (18). These results strongly suggest that the OSE approach, utilizing a single incision and a more muscle-splitting-like access, minimizes dissection and retraction of the paraspinal muscles and soft tissues, thereby reducing postoperative pain and stress response, and ultimately enabling faster patient turnover. This aligns perfectly with the principles of Enhanced Recovery After Surgery (19).

Regarding clinical functional outcomes, our findings provide compelling evidence (20). Postoperative follow-up showed that both groups experienced significant improvement in low back and leg pain VAS scores compared to preoperative levels, with no statistical difference at any time point. This indicates that both techniques were equally thorough and effective in addressing the root cause of pain (nerve compression). However, the OSE group demonstrated a clear advantage in the ODI, which assesses the quality of functional recovery. Patients in the OSE group had significantly lower ODI scores at 1 week, 1 month, and the final follow-up compared to the UBE group. The ODI score comprehensively reflects the impact of pain on a patient's activities of daily living (such as walking, sitting, lifting, and sleeping). This finding suggests that although patients in both groups perceived similar pain intensity, those in the OSE group experienced less difficulty returning to normal life rhythms and performing daily functions, indicating a higher quality of recovery. We hypothesize that this is directly related to the reduced iatrogenic injury to the paraspinal stabilizer muscles, particularly the multifidus, afforded by the OSE technique. Better preservation of muscular architecture provides superior dynamic lumbar stability in the early postoperative period, thereby allowing patients to feel more confident and comfortable during functional activities (21). This advantage is of paramount clinical importance for patients eager to resume work and social activities promptly.

In terms of safety, the incidence and spectrum of complications (including common endoscopic complications such as dural tear and postoperative radiculitis) were not statistically different between the groups, with no serious adverse events such as severe neurovascular injuries recorded in either cohort. This finding is consistent with the conclusions of Previous studies, collectively confirming that the OSE technique, when applied to appropriate indications and performed standardly, possesses a safety profile comparable to that of the established UBE technique (22).

The observed difference in ODI scores between the two groups at final follow-up was statistically significant but below the generally accepted MCID threshold of 10–12 points for ODI in lumbar degenerative diseases (23, 24). This suggests that while the OSE technique offers a measurable advantage in functional recovery, the absolute magnitude of this advantage from the patient's perspective may be modest. However, the significantly faster recovery trajectory (as evidenced by the significant group-by-time interaction) during the early postoperative period (1 week and 1 month) may be of greater clinical relevance to patients, as early functional recovery is often prioritized by those seeking to return to work and daily activities promptly. Future studies with larger sample sizes are needed to confirm whether the observed ODI differences reach the MCID threshold in specific patient subgroups.

Nevertheless, this study has several limitations. First, it was a single-center study, and a larger sample size is needed to enhance the generalizability of the conclusions. Second, the gold standard for assessing surgical invasiveness—postoperative MRI analysis—was only performed in the early postoperative period. There is a lack of assessment of long-term paraspinal muscle changes (such as fatty infiltration, atrophy) and their correlation with long-term clinical outcomes. Third, the study did not employ more objective measures of muscle function (e.g., muscle strength testing). Future research should involve multi-center, large-sample, long-term follow-up studies, incorporating advanced imaging techniques (such as diffusion tensor imaging) and objective functional assessments to gain deeper insight into the long-term impact of different minimally invasive techniques on the spinal stabilizing unit. Fourth, as a retrospective study, it is inherently subject to selection bias, which may affect the generalizability of our findings. Fifth, although we included two distinct pathologies (LDH and LSS), the sample size was insufficient for a well-powered subgroup analysis to determine if the benefits of OSE over UBE are consistent across these conditions. We provided only descriptive data for these subgroups. Larger multi-center studies are needed to confirm the applicability of OSE to specific LDD subtypes. Sixth, although we performed multivariate analysis to adjust for known confounders, the possibility of residual confounding from unmeasured variables (e.g., detailed anatomical severity, patients' occupational demands) cannot be entirely excluded.

In summary, this rigorous comparative study confirms that both OSE and UBE are effective and safe minimally invasive options for treating LDD. Although the OSE technique requires a longer operative time, its optimized surgical access achieves pain relief and haemostatic effectiveness equivalent to UBE, while simultaneously demonstrating clear advantages in promoting faster patient recovery (shorter hospital stay), achieving ultimate minimally invasive appearance (smaller incision), and yielding superior early to mid-term functional recovery quality. Therefore, the OSE technique represents a promising minimally invasive endoscopic innovation that truly embodies the core principle of minimally invasive surgery: achieving the best clinical outcomes with the least trauma. It offers clinicians an excellent option to consider within personalized treatment strategies.

## Data Availability

The raw data supporting the conclusions of this article will be made available by the authors, without undue reservation.
